# Characterization of NanR Regulation of Sialidase Production, Sporulation and Enterotoxin Production by *Clostridium perfringens* Type F Strains Carrying a Chromosomal Enterotoxin Gene

**DOI:** 10.3390/toxins14120872

**Published:** 2022-12-13

**Authors:** Jihong Li, Eric Mi, Arhat Pradhan, Bruce A. McClane

**Affiliations:** Department of Microbiology and Molecular Genetics, University of Pittsburgh School of Medicine, Pittsburgh, PA 15219, USA

**Keywords:** *C. perfringens*, type F food poisoning strains, *nanR*, sialidases, *C. perfringens* enterotoxin and sporulation

## Abstract

*Clostridium perfringens* type F food poisoning (FP) strains produce *C. perfringens* enterotoxin (CPE) to cause a common bacterial food-borne illness in the United States. During FP, CPE is synthesized in the intestines when *C. perfringens* sporulates. Besides CPE, FP strains also produce sialidases. Most FP strains carry their *cpe* gene on the chromosome and all surveyed chromosomal *cpe* (c-*cpe*) FP strains produce NanH sialidase or both NanJ and NanH sialidases. NanR has been shown previously to regulate sialidase activity in non-FP strains. The current study investigated whether NanR also regulates sialidase activity or influences sporulation and CPE production for c-*cpe* FP strains SM101 and 01E809. In sporulation medium, the SM101 *nanR* null mutant showed lower sialidase activity, sporulation, and CPE production than its wild-type parent, while the 01E809 *nanR* null mutant showed roughly similar sialidase activity, sporulation, and CPE production as its parent. In vegetative medium, the *nanR* null mutants of both strains produced more spores than their parents while NanR repressed sialidase activity in SM101 but positively regulated sialidase activity in 01E809. These results demonstrate that NanR regulates important virulence functions of c-*cpe* strains, with this control varying depending on strain and culture conditions.

## 1. Introduction

*Clostridium perfringens* produces at least 21 different toxins, many of which contribute to pathogenicity [1,2,3,4]. However, toxin production varies considerably among different *C. perfringens* isolates. Therefore, carriage of genes encoding six toxins (alpha, beta, epsilon, iota, enterotoxin, and NetB) is used to type *C. perfringens* isolates into seven toxin types (A to G) [5]. By definition, *C. perfringens* type F FP strains must carry the *cpa* and *cpe* genes encoding alpha toxin and enterotoxin (CPE), respectively [5]. CPE production is necessary for the gastrointestinal virulence of type F strains in animal disease models [6] and, very likely, during human disease. There are about 1 million cases/year of *C. perfringens* type F FP in the USA, causing more than USD 310 million in annual economic losses [7,8]. In the elderly or debilitated, this FP can be fatal [9]. Additionally, it is thought that otherwise healthy people with transient predisposing conditions (such as severe constipation) at the time of infection can absorb CPE from the intestines into their blood, causing a lethal enterotoxemia [10,11,12]. Animal model studies suggest this enterotoxemia involves damage to organs such as the heart and kidney, which results in a hyperpotassemia that likely triggers cardiac arrest [13,14]. Type F strains also cause many cases of human non-food-borne gastrointestinal diseases, such as antibiotic-associated diarrhea [15]. 

CPE, a 35 kDa pore-forming toxin, is produced during disease when type F strains sporulate in the intestinal lumen [9,16]. Type F strains produce CPE from either a chromosomal (c-*cpe*) or plasmid-borne (p-*cpe*) *cpe* gene [17,18,19,20]. Most type F FP isolates carry a c-*cpe* gene and those c-*cpe* FP strains are genetically distinct from other *C. perfringens* strains, including the type F strains carrying a p-*cpe* gene that cause non-food-borne gastrointestinal disease, e.g., unlike p-*cpe* type F strains, nearly all type F c*-cpe* strains lack the *pfoA* gene encoding perfringolysin O [20,21]. Genomic diversity amongst type F c-*cpe* strains is also now becoming appreciated [22]. 

In addition to toxins, *C. perfringens* produces a vast array of extracellular enzymes. For example, most *C. perfringens* isolates produce three different sialidases, named NanJ, NanI, and NanH [23]. Both NanJ and NanI are secreted, but NanH remains cytoplasmic during logarithmic vegetative growth [23]. However, at the end of sporulation, NanH is released into culture supernatants when the mother cell lyses to release its mature spore [24]. 

Considerable diversity in sialidase gene carriage exists among type F strains. Similar to many other *C. perfringens* isolates, type F p*-cpe* isolates usually carry the *nanI* gene, typically in combination with the *nanH* and *nanJ* genes [25,26]. In contrast, type F c-*cpe* isolates almost always lack the *nanI* gene [24,25]. However, type F c-*cpe* isolates do carry either the *nanH* gene alone or both the *nanH* and *nanJ* genes [24,25]. 

Sialidases are now emerging as important contributors to *C. perfringens* virulence. Recent studies demonstrated that NanI increases the adherence of NanI-producing *C. perfringens* strains, including type F p*-cpe* strains, to human enterocyte-like cells in vitro and to mouse intestine in vivo [25,26,27,28,29,30]. This result is intriguing since type F p*-cpe* strains cause CPE-associated non-food-borne diarrhea that is often chronic (lasting up to several weeks), while type F c-*cpe* strains lacking *nanI* cause an acute diarrhea lasting less than 24 h [9,15], i.e., colonization promoted by NanI appears to contribute to the chronic nature of diseases caused by p-*cpe* type F strains. NanI also enhances CPE activity, particularly in the presence of mucus [29,30]. Similarly, recent studies demonstrated that NanH also enhances CPE action on cultured Caco-2 cells by increasing the binding of this toxin to receptors on those cells [24]. 

*C. perfringens* can grow using sialic acid generated from sialyl conjugates by the action of sialidases [31,32]. *C. perfringens* growth using sialic acid also involves the *nan* operon, which encodes proteins necessary for the uptake and metabolism of sialic acid [31,32]. Another protein encoded by the *nan* operon is NanR, a member of the RpiR family of transcriptional regulators [32]. NanR was shown to be an important regulator of *nanI* gene expression for NanI-positive type F p-*cpe* strain F4969, where NanR represses *nanI* expression during vegetative growth in TH medium [31]. Furthermore, in modified Duncan-Strong sporulation (MDS) medium, an isogenic *nanR* null mutant of F4969 displayed decreased sporulation and CPE production, yet more NanI production, compared to its wild-type parent [33]. This F4969 phenotype appears to be attributable to the *nanR* mutation since a reversed mutant, where the inserted intron was partially removed from *nanR* mRNA by LtrA, showed increased sporulation and CPE production, but less NanI production, than the *nanR* mutant when cultured in MDS [33]. The *nanR* null mutant of F4969 also exhibited more sustained growth in MDS under the culture conditions used in that study [33]. 

Because of the substantial genomic differences, including variations in sialidase gene carriage, between *C. perfringens* type F p-*cpe* and type F FP c-*cpe* strains, the current study evaluated whether NanR regulates sialidase production, sporulation, and CPE production by type F c-*cpe* FP strains lacking the *nanI* gene but carrying either the *nanH* gene alone or both the *nanH* and *nanJ* genes [24,25].

## 2. Results

### 2.1. Construction and Characterization of SM101 and 01E809 nanR Null Mutants and Reversed Mutants

Previous studies showed that, in type F p-*cpe* strain F4969, NanR regulates expression of *nanI* and several *nan* operon genes encoding proteins involved in sialic acid transport and metabolism [31,33]. To evaluate whether NanR also controls *nanJ* or *nanH* expression in type F c-*cpe* FP strains naturally lacking the *nanI* gene [24,25], *nanR* null mutants of type F c-*cpe* strains SM101 (which carries the *nanH* gene but not the *nanJ* or *nanI* genes [24]) or 01E809 (which carries both the *nanH* and *nanJ* genes but not the *nanI* gene [24]) were constructed using *Clostridium*-modified TargeTron technology [34]. For this purpose, the previously prepared [31] pJIR750*nanR*i vector was electroporated into type F FP wild-type strains SM101 or 01E809. 

**Figure 1:**
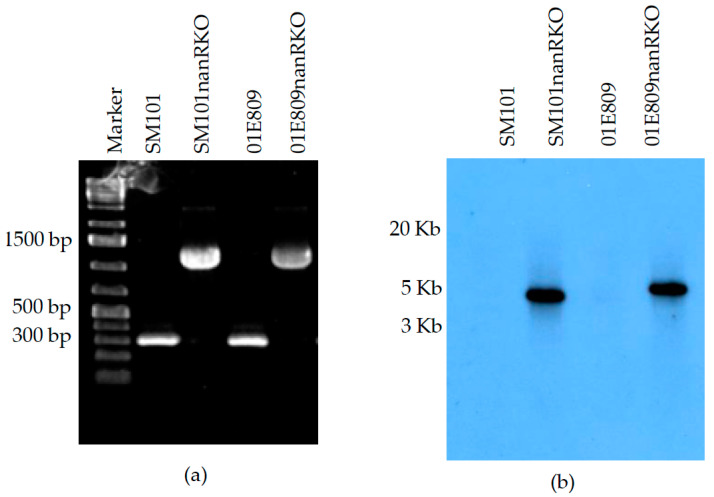
Intron-based mutagenesis to create SM101 and 01E809 *nanR* null mutants and characterization of those mutants by PCR and an intron-specific Southern blot. (**a**) PCR analysis using *nanR* internal primers for wild-type and *nanR* null mutant strains. Because no intron insertion was present, the *nanR* PCR product amplified from wild-type strains was ~300 bp. However, due to insertion of an ~900 bp intron into *nanR,* the PCR product amplified from the *nanR* null mutant strain was ~1200 bp using the same primers. Size of DNA is shown at left. (**b**) Southern blot analysis of an intron-specific probe using DNA from wild-type parents or their *nanR* null mutants. DNA from both wild-type parents and *nanR* null mutants was digested with EcoRI, electrophoresed on a 1% agarose gel prior, blotted, and hybridized with an intron-specific probe. Size of DNA fragments, in kb, is shown at left.

Verifying these mutations, PCR using *nanR* primers amplified a ~300 bp product using DNA isolated from either SM101 or 01E809. However, due to insertion of the ~900 bp intron, PCR with the same primers amplified a 1200 bp product from both null mutant strains (Figure 1a). To confirm that only a single intron had been inserted into each *nanR* null mutant, a Southern blot was performed using an intron-specific probe. Results demonstrated (Figure 1b) the presence of only one intron insertion in both the SM101 and 01E809 *nanR* null mutants. These *nanR* null mutants were named SM101nanRKO and 01E809nanRKO. 

It is difficult to complement the *nanR* mutants by cloning *nanR* into a shuttle plasmid because *nanR* is part of a large operon [32,35,36]. Therefore, to address whether *nanR* mutant phenotypes might be merely due to spontaneous secondary mutations, our previous study with F4969 reversed the *nanR* mutation. This reversal was possible because the F4969 *nanR* null mutant has a group II intron inserted into its *nanR* target gene in a sense orientation [31]. Therefore, transformation of the pJIR750nanRi vector, which encodes LtrA, back into the *nanR* null mutant created a reversed mutant whereby LtrA splices out the intron from some intron-disrupted *nanR* mRNA to restore partial production of wild-type mRNA. 

A similar approach was used in the current study for the SM101 and 01E809 *nanR* null mutants. Those reversed mutants are named SM101nanRKOrev and 01E809nanRKOrev.

### 2.2. nanR Is Expressed When SM101 and 01E809 Are Cultured in Either TH Vegetative Medium or MDS Sporulation Medium

**Figure 2:**
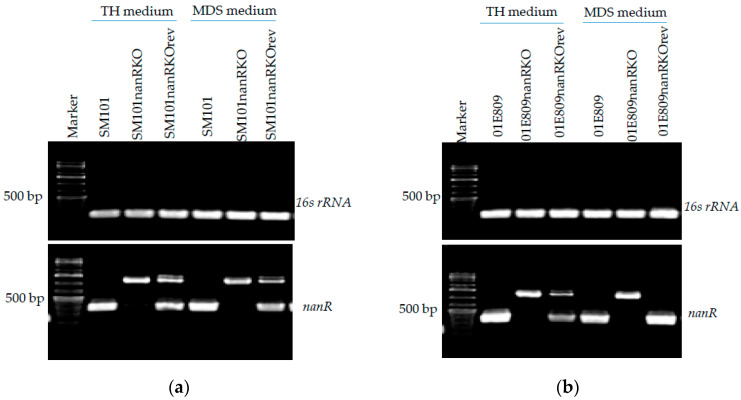
RT-PCR analyses of *nanR* gene transcription in type F parent strains and their *nanR* null mutants or reversed mutants in both TH and MDS. RT-PCR analysis showing *nanR* expression by (**a**) SM101, its *nanR* null mutant, and a reversed mutant and (**b**) 01E809, *nanR* null mutant, and reversed strains. All strains were cultured for 4 h at 37 °C. Size of DNA is shown at left. 16S rRNA RT-PCR was used as a house-keeping gene control for transcription. Results shown are representative of three repetitions.

TH is generally considered a vegetative growth medium for *C. perfringens*, while MDS is a sporulation medium. To assess *nanR* expression by c*-cpe* type F stains in these media, *nanR* RT-PCR was performed on SM101 or 01E809 and their null mutant strains or reversed mutants cultured in TH or MDS. RT-PCR results for SM101 and derivatives are shown in Figure 2a, while RT-PCR results for 01E809 and its derivatives are shown in Figure 2b. In addition, PCR without RT for the *polC* house-keeping gene confirmed that all RNA samples were DNA-free (data not shown), while RT-PCR for 16S rRNA gene expression confirmed the quality of all RNA preparations. 

Figure 2 results show a *nanR* RT-PCR amplified ~300 bp product using cDNA prepared from both wild-type parents and their reversed mutants, whether cultured in TH or MDS, confirming expression of wild-type *nanR* mRNA by these strains under both culture conditions. However, the *nanR* null mutants did not amplify this 300 bp product, confirming that they do not express wild-type *nanR* mRNA in either TH or MDS. The larger 1200 bp band amplified for the mutants, and some reversed mutant, represents *nanR* mRNA with an intron insertion.

### 2.3. Comparison of Growth of Wild-Type Parent Strains, Their nanR Null Mutants and Their Reversed Mutants in TH and MDS Medium

A previous study [31] reported that type F p-*cpe* strain F4969, an isogenic *nanR* null mutant strain, and a reversed mutant all grew similarly in TH medium when cultured at 30 °C. However, that same F4969 *nanR* null mutant grew slower when cultured at 30 °C in MDS compared to its wild-type parent and reversed mutant strain [33]. Those studies had to use 30 °C incubation because the F4969 reversed mutant showed poorly or unrestored expression of wild-type *nanR* mRNA at 37 °C (data not shown). 

**Figure 3:**
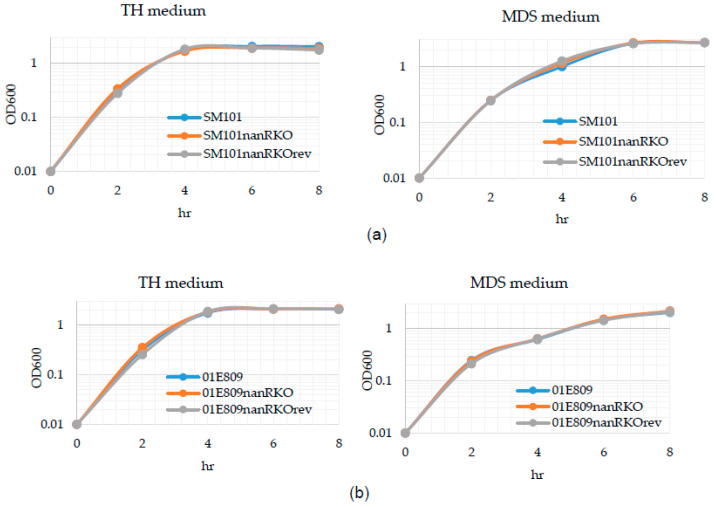
Post-inoculation changes in the OD_600_ for SM101 and 01E809, as well as their *nanR* null mutants or reversed mutants, when cultured in TH or MDS. OD_600_ changes for (**a**) SM101, its *nanR* null mutant, and reversed mutant and (**b**) 01E809, its *nanR* null mutant, and reversed mutant. Each strain was cultured in TH or MDS at 37 °C. Every 2 h up to 8 h, a 1-mL aliquot of the culture was removed and the OD_600_ was determined. Results shown are representative of three repetitions.

In the current study, wild-type SM101 did not produce either sialidase activity or CPE when cultured in MDS at 30 °C (data not shown). Considering those observations, the Figure 2 results indicating that the *nanR* mutation was reversed well at 37 °C for both SM101 and 01E809, and the pathophysiological relevance of 37 °C for type F disease, all subsequent experiments in the current study were performed at 37 °C. The current study determined that wild-type SM101 and 01E809 grew similarly as their isogenic *nanR* null mutants or their reversed mutants when cultured in either TH or MDS at 37 °C (Figure 3). 

### 2.4. NanR Regulates the Sialidase Activity of Type F FP c-cpe Strains SM101 and 01E809

A previous study [24] determined that SM101 produces NanH only during sporulation, with NanH then accumulating intracellularly until being released, along with CPE, when the mother cell lyses to release its mature spore. To confirm that previous result [24], and also compare when sialidases are produced and released by a type F c-*cpe* strain that (unlike SM101) encodes both *nanH* and *nanJ*, the current study conducted a head-to-head comparison of the sialidase activities of culture supernatants (which contain only extracellular sialidase activity) and sonicated whole cultures (which contain both intracellular and extracellular sialidase activity) at three time points (4 h, 8 h, or 24 h) for SM101 vs. 01E809 cultured in TH or MDS. In addition, sialidase activities of SM101 or 01E809 *nanR* null mutants and reversed mutants were also assessed in TH and MDS cultures to compare NanR regulatory effects in these c-*cpe* strains, if any. 

**Figure 4:**
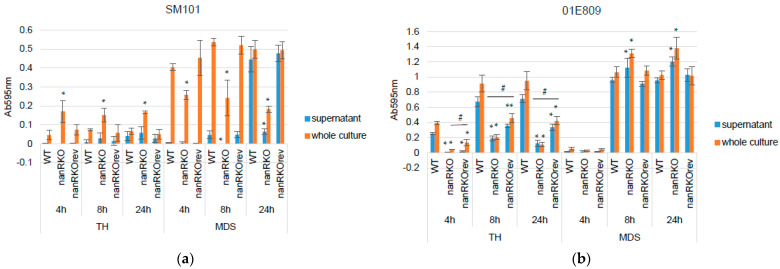
Comparison of sialidase enzyme activity in TH or MDS culture supernatants and sonicated whole cultures. Sialidase activity shown for (**a**) wild-type SM101, its *nanR* null mutant, and the reversed mutant or (**b**) wild-type 01E809, its *nanR* null mutant, and a reversed mutant. The samples were collected at 4 h, 8 h, and 24 h in cultures incubated at 37 °C. Orange bars show sonicated whole culture sialidase enzyme activity. Blue bars show culture supernatant sialidase enzyme activity. Shown are the mean values from three independent experiments. The error bars indicate the S.D. * *p* < 0.05 relative to wild-type. # *p* < 0.05 relative to *nanR* null mutant.

Sialidase activity results (Figure 4a) showed that, in TH medium, wild-type SM101 and its *nanR* reversed mutant produce low sialidase activity, even when using whole (sonicated) 24 h cultures. However, whole (sonicated) TH cultures of the *nanR* null mutant strain had significantly greater sialidase activity compared to whole (sonicated) TH cultures of wild-type SM101 at all three time points. Indicative of substantial intracellular NanH, only limited sialidase activity was present in the TH culture supernatants of the *nanR* null mutant at any timepoint.

In MDS, sialidase activity was mainly present in whole (sonicated) cultures of SM101 at 4 or 8 h, but significant sialidase activity became detectable in 24 h culture supernatants. Furthermore, there was significantly more sialidase activity present in whole (sonicated) MDS cultures of SM101 compared to whole TH cultures of this same strain at all timepoints. In MDS cultures, sialidase activity in whole (sonicated) cultures of the SM101 *nanR* null mutant was significantly decreased at all three timepoints compared to the sialidase activity in MDS whole (sonicated) cultures of the wild-type strain or the reversed mutant. In 24 h MDS cultures of wild-type SM101 or the reversed mutant, nearly similar sialidase activity was present in culture supernatants and the whole (sonicated) culture. Collectively, these findings confirm previous results for SM101 indicating that NanH is made preferentially in sporulating cultures and released upon lysis of the mother cells [24].

While the only sialidase gene carried by SM101 is *nanH*, 01E809 possesses both the *nanJ* and *nanH genes* [24]. Given this difference in sialidase gene carriage, sialidase activity was also measured at three time points (4 h, 8 h, or 24 h) for whole (sonicated) cultures or culture supernatants for 01E809, its *nanR* null mutant, or reversed mutant grown in TH or MDS. The results (Figure 4b) showed that, relative to TH cultures of SM101, TH cultures of 01E809 contained more sialidase activity in culture supernatants, with this activity becoming detectable by 4 h and then increasing throughout the 24 h of culture. Furthermore, sialidase activity was only slightly higher in sonicated (whole) cultures vs. culture supernatants of 01E809 when cultured in TH. However, for TH cultures of the 01E809 *nanR* null mutant, sialidase activity in culture supernatants or whole (sonicated) cultures was significantly lower compared to the wild-type parent at all three time points. Consistent with the reversed mutant only partially restoring NanR expression, the supernatant or whole (sonicated) culture sialidase activity of this strain grown in TH was significantly less than for its wild-type parent, yet significantly higher than for the *nanR* null mutant, at all three timepoints. 

For MDS cultures, little or no sialidase activity was detected for 01E809 or its derivatives in 4 h cultures, although 8 h or 24 h cultures of 01E809 and its derivatives all showed strong sialidase activity in both supernatants and whole (sonicated) cultures, with only slightly higher sialidase activity present in the sonicated cultures. In contrast to MDS cultures of SM101 and its derivatives, sialidase activity was slightly higher in both culture supernatants and whole (sonicated) cultures of the 01E809 *nanR* null mutant vs. its wild-type parent or the reversed mutant grown in MDS. These results are indicative of NanR repression of total sialidase activity when 01E809 is cultured in MDS. 

**Figure 5:**
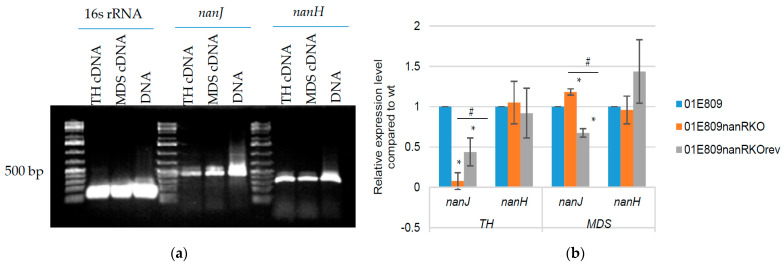
RT and qRT-PCR analyses of *nanJ* and *nanH* gene transcription. (**a**) RT-PCR analyses of *nanJ* and *nanH* gene expression in wild-type 01E809 cultured for 4 h at 37 °C in TH or MDS medium. 16S rRNA RT-PCR was used as a house-keeping gene control for transcription. 01E809 DNA served as a positive control. DNA markers were loaded for RT-PCR of two genes. Results shown are representative of three repetitions. (**b**) qRT-PCR analyses of expression of the *nanJ* and *nanH* genes in TH and MDS cultures of 01E809. RNA samples were isolated from cells grown for 4 h at 37 °C in the specified medium. Twenty nanograms of RNA were used to determine transcript levels. Average C_T_ values were normalized to those of the house-keeping 16S rRNA gene and the comparative C_T_ method (2^−ΔΔCT^) was used to calculate fold differences. The value of each bar depicts the calculated fold change relative to the control. Shown are the mean values from three independent experiments. The error bars indicate the S.D. * *p* < 0.05 relative to wild-type. # *p* < 0.05 relative to *nanR* null mutant.

Since 01E809 produces both NanJ and NanH, experiments directly assessed whether NanR regulates expression of *nanJ* or *nanH* (or both genes) when this strain is cultured in TH or MDS. RT-PCR confirmed *nanJ* and *nanH* expression by wild-type 01E809 in both TH and MDS (Figure 5a). Using cDNA from both 01E809 and the *nanR* null mutant, qRT-PCR then showed that, in MDS, there was more *nanJ* expression for the *nanR* null mutant vs. wild-type 01E809. However, in TH, *nanJ* gene expression was lower for the *nanR* mutant than for its wild-type parent (Figure 5b). Both differences were statistically significant. The qRT-PCR results also confirmed that the reversed mutant exhibits partially restored *nanJ* expression when cultured in TH and MDS.

Compared to wild-type 01E809, *nanH* expression by the *nanR* null mutant was slightly higher in TH but a little less in MDS, although those differences did not achieve statistical significance (Figure 5b). 

### 2.5. NanR Regulates Sporulation and CPE Production by SM101 or 01E809 When Cultured in TH or MDS

Our previous study showed that NanR positively regulates both sporulation and CPE production in MDS cultures of type F p-*cpe* strain F4969 [33]. The current study next sought to determine whether NanR also regulates sporulation and CPE production for the type F c-*cpe* strains SM101 and 01E809. 

**Figure 6:**
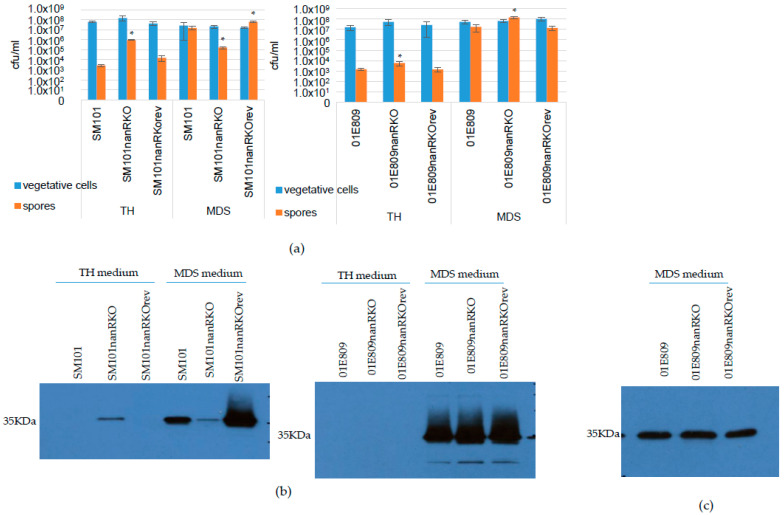
Comparison of viable vegetative cells and heat-resistant spore formation, or CPE production, in TH and MDS cultures of type F strains and their derivatives. (**a**) Vegetative cells and heat-resistant spore formation levels for SM101 (left panel) or 01E809 (right panel) and their *nanR* null mutant strains or their *nanR* reversed mutants. The bacteria were grown in TH or MDS cultures for 24 h at 37 °C, and, after a 10-fold serial dilution with PBS buffer, then plated onto BHI agar plates and grown anaerobically overnight at 37 °C for colony counting of viable vegetative cells. An aliquot of each culture was then heat-shocked for 20 min at 70 °C before plating, with the resultant colony counts representing germinated heat-resistant spores. Shown are the mean values from three independent experiments. The error bars indicate the S.D. * *p* < 0.05 relative to wild-type. (**b**) Western blot analyses of CPE production by SM101 (left panel) or 01E809 (right panel), their *nanR* null mutants, or *nanR* reversed mutants using TH or MDS 24 h culture supernatants. (**c**) Western blot analyses of CPE production by same sample of 01E809 shown in (**b**) except for a 20-fold dilution. Migration of a 35 kDa protein (the size of CPE) is shown at left. Shown is a representative blot of three repetitions.

In either TH or MDS, the same number of viable vegetative cells were present in 24 h cultures of SM101 vs. its derivatives (Figure 6a). Similarly, there were no differences in the number of viable vegetative cells present in 24 h TH or MDS cultures of 01E809 vs. its derivatives (Figure 6a). Interestingly, after the same 24 h incubation in this vegetative growth medium, TH cultures of all six strains also contained at least low levels (10^3^/mL) of heat-resistant spores (Figure 6a). Moreover, after a 24 culture in TH, the *nanR* null mutants of both SM101 and 01E809 produced significantly more spores compared to their wild-type parents or reversed mutants (Figure 6a). After 24 h incubation in TH, the SM101 *nanR* null mutant even made sufficient numbers (~10^6^/mL) of spores to allow Western blot detection of CPE production, which is sporulation dependent (Figure 6b).

In MDS medium, the SM101 *nanR* null mutant produced significantly less spores compared to wild-type SM101 or the reversed mutant (Figure 6a). Consistent with that result, the SM101 *nanR* null mutant also made less CPE when cultured in MDS (Figure 6b).

Interestingly, MDS cultures of the *nanR* null mutant of 01E809 contained a modest, but significant, increase in spore numbers compared to its wild-type parent or reversed mutant. However, the MDS cultures of the 01E809 *nanR* mutant did not contain significantly more CPE (Figure 6b,c) and qRT-PCR for *cpe* gene expression did not detect any significant differences between 4 h MDS cultures of wild-type 01E809 and its *nanR* null mutant (data not shown). Those results support NanR having limited effects on CPE production for this c-*cpe* strain when cultured in MDS.

## 3. Discussion

*Clostridium perfringens* strains produce up to three different sialidases (NanJ, NanI, and NanH) but this varies considerably amongst different strains [23,25,37]. *C. perfringens* type F c-*cpe* strains, which are the major cause of type F FP, often lack the *nanI* gene but consistently carry the *nanH* gene and, sometimes, also carry the *nanJ* gene [24,25]. Previous studies demonstrated that NanR can regulate sialidase activity of type F p-*cpe* strain F4969 [31,33], but NanR regulation of sialidase activity of type F c-*cpe* strains had not yet been evaluated prior to the current study. 

Specifically, the previous study reported that a *nanR* null mutant of type F p-*cpe* strain F4969 produced significantly more total (secreted and intracellular) sialidase activity than its parent when cultured in MDS or TH, indicating that NanR represses sialidase production by F4969 under these culture conditions [31,33]. This repression was shown to involve NanR decreasing expression of the *nanI* gene, with little or no effect on expression of the *nanJ* or *nanH* genes by F4969 [33]. When the current study tested whether NanR regulates total sialidase production by two c-*cpe* FP strains, i.e., SM101 and 01E809, the results revealed that NanR also regulates total sialidase production by these two type F strains. However, the regulatory pattern varied between these c-*cpe* strains and F4969. 

The current study demonstrated that, like F4969, NanR represses total sialidase activity in TH cultures of SM101. However, unlike F4969, NanR increased total sialidase activity in SM101 MDS cultures. Since SM101 carries only the *nanH* gene, these results establish that NanR can also regulate *nanH* expression in some type F strains and that NanR can also promote sialidase expression, i.e., NanR regulation can be either positive or negative depending upon the strain and culture conditions. Moreover, the current study also revealed that the pattern by which NanR regulates total sialidase production can vary among type F c-*cpe* strains in the same culture conditions. Specifically, NanR had opposite regulatory effects in 01E809 from those observed for SM101, i.e., for 01E809, NanR increased total sialidase activity in TH cultures but repressed sialidase activity in MDS. 

Since 01E809 carries two sialidase genes [24], the current study then assessed which sialidase gene(s) of this strain are regulated by NanR in TH or MDS cultures. RT-PCR showed that both sialidase genes are expressed when 01E809 is cultured in either media. qRT-PCR then revealed that NanR has no significant regulatory effect on *nanH* expression when 01E809 is cultured in either TH or MDS. In contrast, NanR regulated *nanJ* expression in both media, although the effects were opposite, i.e., for 01E809, NanR increased *nanJ* expression in TH cultures but repressed *nanJ* transcription in MDS cultures. Collectively, these studies strongly suggest that NanR controls the total sialidase activity of 01E809 primarily by regulating *nanJ* expression. 

Combining the current results for 01E809 and SM101 with the previous F4969 results [31,33], it becomes apparent that NanR can regulate expression of all three *C. perfringens* sialidases, but the nature of this regulation is highly strain- and culture condition-variable. The basis for this variability requires further study. However, one potentially interesting pattern is that among the three type F strains studied to date, NanR regulates expression of the gene encoding the sialidase predominantly responsible for that strain’s sialidase activity, i.e., NanR regulates expression of *nanI* in F4969 [31,33], and as shown in the current study, *nanH* in SM101, and *nanJ* in 01E809 (evidence that NanJ is the predominant sialidase of 01E809 is presented below). Future studies should evaluate whether this relationship holds for other type F strains or other types of *C. perfringens* isolates. 

This study is also informative regarding production of the individual sialidases. A previous study reported that SM101 produces much more NanH in MDS vs. TH, which was confirmed in the present study, i.e., MDS cultures of SM101 had much higher sialidase activity than TH cultures of this strain [24]. Consistent with the absence of a secretion signal, NanH was reported to accumulate intracellularly inside of sporulating SM101 cells until being released by mother cell lysis, which takes > 8 h. The present results also confirm that finding for SM101, i.e., much higher sialidase activity was measured in whole MDS cultures of this strain vs. culture supernatants up to 8 h but substantial supernatant sialidase activity became detectable by 24 h, a time by which substantial mother cell lysis has occurred [24].

Prior to the current work, there had been only limited study of NanJ-producing type F strains. The current study demonstrated that 01E809 expresses both the *nanH* and *nanJ* genes in either 4 h TH or MDS cultures. This study also showed 01E809 produces much higher total culture sialidase activity than SM101 whether grown in TH or MDS medium, suggesting that NanJ could be a major contributor to 01E809 whole culture sialidase activity. Supporting this possibility was the observation that the *nanR* mutant of 01E809 produced much less total sialidase activity in TH; that result appears informative because, as already discussed, qRT-PCR indicated that NanR regulates *nanJ* but not *nanH* expression in TH cultures of 01E809. Additional support for NanJ being the major contributor to 01E809 total sialidase activity includes determining that, unlike 4 or 8 h TH or MDS cultures of SM101 (which lacks *nanJ*), the great majority of total sialidase activity in 01E809 TH or MDS cultures was present in the supernatants of 4 h or 8 h TH cultures or 8 h MDS cultures. This finding is consistent with NanJ involvement in the higher sialidase activity of 01E809 vs. SM101 cultures since NanJ has a secretion signal and mother cell lysis (as needed for NanH release) does not occur by 8 h. NanJ contributions to c-*cpe* type F FP strain sialidase activity will be examined more closely in future studies. 

NanR was previously shown to increase sporulation and CPE production in MDS cultures of F4969 [33]. Therefore, the current study also evaluated the effects of NanR on growth, 24 h vegetative cell viability, sporulation, and CPE production of type F c-*cpe* strains. For either SM101 or 01E809, NanR did not significantly affect culture growth or 24 h vegetative cell viability in either TH or MDS cultures. Combining this observation with the finding that NanR does regulate sialidase production by these two strains suggests NanH (for SM101) or NanH and NanJ (for 01E809) are not important for vegetative cell growth or survival of these c-*cpe* strains in these two media. 

Since TH is usually considered a vegetative growth medium, it was interesting that 24 h TH cultures of both wild-type parents and all derivatives contained at least 10^3^ spores/mL. However, the *nanR* null mutants of both SM101 and 01E809 contained significantly more spores. For the SM101 *nanR* null mutant, there was sufficient sporulation levels (10^6^ spores/mL) to permit detection of CPE production in 24 h cultures. Presumably, sporulation of all of these strains in TH cultures, which was much less than for corresponding MDS cultures, reflects the harsh environment present after 24 h of culture and involves factors such as starvation and the accumulation of toxic wastes. It is possible that the increased sialidase activity in 24 h TH cultures of the SM101 *nanR* mutant contributed to the increased sporulation of this strain by generating nutrients to complete sporulation, but this would require further study. Even if true, that relationship clearly does not hold true for TH cultures of the 01E809 *nanR* null mutant which contain less sialidase activity yet sporulates better than its wild-type parent.

As previously noted for F4969 [33], NanR significantly increased sporulation and CPE production when SM101 was cultured in MDS. However, NanR had the opposite effect for 01E809 MDS cultures, where it mildly repressed sporulation. These results further support diversity in gene regulation between these two c-*cpe* strains. 

There remains a clear need for further studies to understand sialidase production by *C. perfringens*. For example, why does this bacterium produce up to three different sialidases, each with different characteristics [37]? Why is there diversity in the number of sialidases produced by different strains? Many *C. perfringens* virulence genes are associated with mobile genetic elements such as plasmids or IS sequences [38,39], but there is no evidence that *C. perfringens* sialidase genes, which are chromosomal, are associated with mobile genetic elements. 

Similarly, it is unclear why NanR regulation of sialidase gene expression varies in different culture conditions or between strains, e.g., *nanH* expression is NanR-regulated in SM101 but not in 01E809 or F4969. This variability is intriguing since (i) the *nanR* sequence is highly conserved (~99% identity) between all sequenced *C. perfringens* strains, including SM101 and F4969, and (ii) sequences upstream of the *nanH* ORF are highly conserved (~97% identity) between SM101 and F4969, yet *nanH* expression is NanR-regulated in SM101 but not F4969. One clue to these regulation differences might involve the presence upstream of sialidase ORFs of putative regulatory sites for several other regulators, such as CodY, CcpA, and Spo0A, in addition to NanR [24,40]. Like NanR, many of these other regulators are sensitive to nutritional conditions, which could help explain the differences in sialidase gene expression in different culture conditions. The involvement of other regulatory factors may also explain why NanR exhibits only partial regulatory effects on sporulation and CPE production for both c-*cpe* strains in the current study. Supporting that possibility, the global regulatory protein CodY resembles NanR by also promoting sporulation and CPE production when SM101 is cultured in MDS [41]. Future studies should further examine the interplay between these regulatory proteins to obtain a more complete picture of how c-*cpe* strains regulate sialidase production, sporulation, and CPE production and why this regulation differs amongst c-*cpe* strains and in different culture conditions. 

Considering the above discussion, our current working model for sialidase gene regulation involves a complex interplay between NanR and several other nutritionally sensitive regulators. Differences in expression levels of those regulators, or processing and transport of nutrients to affect the activity of these regulators, then leads to variations in regulation of sialidase gene expression among *C. perfringens* strains. Future studies are needed to test this model.

## 4. Materials and Methods

### 4.1. C. perfringens Strains, Plasmid, Media and Chemicals

*C. perfringens* SM101 is a transformable derivative of a c-*cpe* type F isolate obtained from a case of human FP disease in Europe during the 1950s [42]. *C. perfringens* 01E809 is a transformable c-*cpe* type F strain obtained from a case of human FP disease that occurred in the USA during the 1990s [11]. A previously prepared plasmid named pJIR750nanRi [31], which carries a *nanR*-targeted group II intron in the sense orientation, was used in this study to construct SM101 and 01E809 *nanR* null mutants, as well as a *nanR* reversed mutant from the *nanR* null mutants. 

*C. perfringens* stock cultures were prepared using cooked meat medium (CMM, Difco Laboratories, Franklin Lakes, NJ, USA). For vegetative cultures, FTG medium (fluid thioglycolate medium; Difco Laboratories, USA), TH medium (Bacto Todd Hewitt Broth [32], with 0.1% sodium thioglycolate [Sigma Aldrich, St. Louis, MO, USA]), and TGY medium (3% tryptic soy broth [Becton-Dickinson, Franklin Lakes, NJ, USA], 2% glucose [Fisher Scientific, Hampton, NH, USA], 1% yeast extract [Becton-Dickinson, USA], and 0.1% sodium thioglycolate [Sigma Aldrich, USA]) were used. To obtain strongly sporulating cultures, *C. perfringens* strains were grown in MDS sporulation medium (proteose peptone [Becton-Dickinson, USA] 15 g/L, yeast extract [Fisher Scientific, USA] 4 g/L, sodium thioglycolate 1 g/L, disodium phosphate [Fisher Scientific, USA] 10 g/L, raffinose [Fisher Scientific, USA] 4 g/L, and caffeine [Fisher Scientific, USA] 19.2 g/L). For mutant selection and plate counting, BHI agar plates (Brain heart infusion, Becton-Dickinson, USA) with or without chloramphenicol (Cm, from Fisher Scientific, USA) were utilized. To obtain the pJIR750nanRi plasmid, Luria–Bertani (LB) broth (1% tryptone [Becton-Dickinson, USA], 0.5% yeast extract, and 1% NaCl [Fisher scientific, USA]) was used for culture. 

### 4.2. Construction and Characterization of SM101 and 01E809 nanR Null Mutants and Reversed Mutants

The *nanR* gene in both SM101 and 01E809 were inactivated by insertion, in the sense orientation, of a targeted group II intron using the *Clostridium*-modified TargeTron system [34]. For this purpose, the previously prepared pJIR705nanRi plasmid [31] containing an intron targeted for insertion into the *nanR* gene was electroporated into each wild-type strain. Mutants were then selected using BHI agar plates containing 15 mg/L Cm. The *nanR* null mutant primers used for screening were nanRKOF and nanRKOR, which were described previously [31]. The PCR reactions for this screening included 1 µL of each pair of primers (at a 0.5 µM final concentration), 1 µL of purified DNA template (100 ng), and 25 µL of 2× DreamTaq Green PCR Master Mix (Fisher Scientific, USA), which were mixed together before ddH_2_O was added to reach a total volume of 50 µL. The reaction mixtures were placed in a thermal cycler (Techne, Minneapolis, MN, USA) and subjected to the following amplification conditions: 1 cycle of 95 °C for 2 min, 35 cycles of 95 °C for 30 s, 55 °C for 40 s, and 72 °C for 1 min 40 s, and a single extension of 72 °C for 5 min. PCR products were then electrophoresed on a 1.5 % agarose gel, which was stained with ethidium bromide. After curing the pJIR750nanRi plasmid, the resultant mutants were named SM101nanRKO or 01E809nanRKO. 

To construct *nanR* reversed mutants named SM101nanRKOrev or 01E809nanRKOrev, the pJIR750nanRi plasmid was electroporated into each of the *nanR* null mutants. In the current study, growth at 37 °C partially reversed the intron insertion in *nanR* mRNA expressed by nanRrev, presumably by causing Ltr-mediated splicing removal of the intron at the transcriptional level [43]. Expression of some wild-type *nanR* mRNA by the two reversed mutants was confirmed by *nanR* RT-PCR (as described below). 

Southern blot analyses using an intron-specific probe were performed to confirm that both *nanR* null mutants only have a single intron insertion in their genome. An intron-specific probe was prepared by PCR and a DIG labelling kit (Sigma-Aldrich, USA), as previously described [31]. DNA was prepared from wild-type SM101 or 01E809, and their *nanR* null mutant strains, using the Epicentre DNA purification kit (purchased from Fisher Scientific, USA). An aliquot of each purified DNA (3 µg) was then digested overnight at 37 °C with EcoRI, and then electrophoresed on a 1% agarose gel. After digested DNA was alkali-transferred to a nylon membrane (Sigma-Aldrich, USA), the blot was hybridized with a digoxigenin-labeled intron specific probe. DIG detection reagents and CSPD substrate (Sigma-Aldrich USA) were used to detect hybridized probes, according to the manufacturer’s instructions. 

### 4.3. C. perfringens mRNA Isolation, RT and qRT-PCR Analysis

SM101, 01E809, and their *nanR*KO or *nanR*rev derivatives were grown in TH or MDS broth for 4 h at 37 °C. From pelleted cultures, RNA was extracted using saturated phenol and purified by TRIzol and chloroform (Life Technologies and Sigma Aldrich, USA), as previously described [44]. PCR without reverse transcriptase (RT) confirmed that the isolated RNA samples were DNA-free before RT PCR or qRT-PCR analysis. If DNA contamination was detected, DNase (Fisher Scientific, USA) removed the residual DNA contamination. Each purified RNA was then quantified by determining A_260_ and used to prepare cDNA with a Maxima first-strand cDNA synthesis kit (Fisher Scientific, USA), according to the manufacturer’s instructions. For each experiment, *nanR* RT PCR was performed using cDNA from each wild-type parent or its *nanR*KO and *nanR*rev strains, which ensured that the reversed mutant had made wild-type *nanR* mRNA. The *nanJ* and *nanH* primers used for qRT-PCR primers have been described previously [31]. Each cDNA was diluted 10 times to 5 ng/µL. qRT-PCR was performed using Power SYBR green PCR master mix (Fisher Scientific, USA) and a StepOnePlus qRT-PCR instrument (Applied Biosystems, USA), as described in an earlier paper [31]. Following qRT-PCR, relative mRNA expression was normalized to constitutive expression levels of the house-keeping 16S RNA and calculated using the comparative threshold cycle (2^−ΔΔCT^) method [44]. 

### 4.4. Type F Strain Growth Curve Analyses and Quantitative Counts of Viable Vegetative Cells and Heat-Resistant Spores in TH or MDS Cultures

Measurement of growth (OD_600_) and quantitation of viable vegetative cells and heat-resistant spore formation in TH or MDS cultures of type F strain parents or their derivatives were performed, as described previously [44]. For heat-resistant spore enumeration, the cultures were heat-shocked at 70 °C for 20 min to kill the vegetative cells and promote heat-resistant spore germination [44]. 

### 4.5. Measurement of Sialidase Enzyme Activity

To measure sialidase activity, a 0.2 mL aliquot of a FTG overnight culture was transferred to 10 mL of fresh TH or MDS medium and those cultures were then incubated at 37 °C for different times, as indicated (see Results). Some aliquots of these TH or MDS cultures were removed and sonicated with a Qsonica sonicator, USA. The sonication program and the sonicated sample volume were described previously [24]. After removal of aliquots for sonication, the remainder of each culture was centrifuged, and the supernatants were used to determine sialidase activity. An aliquot (60 μL) of supernatant from each sonicated culture or culture supernatant was added to 40 μL of substrate (4 mM 5-bromo-4-chloro-3-indolyl-α-D-N-acetylneuraminic acid (Santa Cruz, Santa Cruz, CA, USA)). The mixture was incubated at 37 °C for 1 h and absorbance at 595 nm was then measured using a Bio-Rad microplate reader. 

### 4.6. CPE Western Blot Analyses

For CPE Western blot analyses, a 0.2 mL aliquot sample from an overnight FTG culture of each type F parent strain and their *nanR* null mutant or reversed mutant were individually inoculated into 10 mL of MDS or TH media for 24 h. To perform a CPE Western blot, culture aliquots were removed, and supernatants were then mixed with 5× SDS loading buffer. The CPE Western blot was then performed using a CPE anti-rabbit polyclonal antibody, as described previously [25].

### 4.7. Statistical Analyses

GraphPad Prism 8 was used to perform statistical analyses. For comparing >2 samples, one-way analysis of variance (ANOVA) was applied with post-hoc analysis by Dunnett’s multiple-comparison test. For comparing two samples, Student’s *t*-test was applied. Differences were considered significant if the *p* value was <0.05.

## Data Availability

Not applicable.
